# What are the perceived unmet needs for patient care, education, and research among genitourinary cancer nurses in Australia? A mixed method study

**DOI:** 10.1016/j.apjon.2024.100564

**Published:** 2024-07-25

**Authors:** Catherine Paterson, Helen Anderson, Michelle Rosano, Donna Cowan, Diana Schulz, Kerry Santoro, Tina Forshaw, Cynthia Hawks, Natasha Roberts, On behalf of the Cancer Nurses Society of Australia

**Affiliations:** a Caring Futures Institute, College of Nursing and Health Sciences, Flinders University, Adelaide, Australia; b Central Adelaide Local Health Network, Adelaide, Australia; c Robert Gordon University, Aberdeen, Scotland, UK; d Cancer, Blood Disorders and Respiratory Service, Gold Coast University Hospital (GCUH), Australia; e Cancer Care Griffith, Australia; f Monash University, Frankston, Australia; g Wide Bay Hospital and Health Service, Queensland, Australia; h Canberra Health Services, Australia; i Fiona Stanley Hospital, Murdoch, WA, Australia; j School of Medicine University of Western Australia, Nedlands, WA, Australia; k STARS Alliance: Metro North Health and the University of Queensland, Australia; l Queensland University of Technology, Australia; m University of Queensland Centre for Clinical Research, Australia

**Keywords:** Genitourinary neoplasms, Oncology nursing, Qualitative research, Needs assessment, Psychological distress, Health priorities

## Abstract

**Objective:**

Specialist genitourinary (GU) nurses provide care to a broad and diverse group of patients diagnosed with kidney, bladder, prostate, testicular, adrenal, and penile cancer. The purpose of this study was to identify GU cancer nurse perspectives of perceived unmet needs in service provision, specific educational and research priorities.

**Methods:**

A concurrent mixed methods study design incorporated quantitative and qualitative data collection from the GU Cancer nurses workforce in Australia. Quantitative data collected using an electronic survey instrument and were analysed using descriptive statistics. Qualitative data collected through semi-structured interviews and coded for thematic analysis. Ethical approval was gained.

**Results:**

Fifty responses were received from the electronic survey. 39/50 (78%) were female and 35 (70%) were metropolitan based. The highest domains of perceived unmet needs related to psychological/emotional needs – 17/23 (74%), intimacy needs – 15/23 (65%) and informational needs – 13/23 (57%). The themes from the qualitative interviews identified: (1) Patient needs – lack of tumour specific contact for cancer patients, fragmented delivery of cancer care, perception of better access to supportive care for public patients, lack of access to supportive care screening tools for needs assessment. (2) Educational needs – lack of GU specific cancer educational resources/learning opportunities and barriers to accessing educational opportunities. (3) Research priorities – impact on carers/partners, specific needs of different GU cancers, future focus on genetic testing/counselling, interventions for financial toxicity and development of models of care for geriatric GU patients.

**Conclusions:**

Specialist GU cancer nurses support a broad group of patients. Given the prominence of addressing unmet cancer care needs among people with GU cancers in this study, cancer nursing as a discipline alongside the multidisciplinary team, requires innovative solutions to overcome fragmented care which is often highly complex, and develop individualised and integrated care across the cancer care continuum. We encourage clinicians, researchers, policy makers, people affected by cancer, and their care networks, to continue to drive innovation by (1) Embedding an integrated approach to cancer nursing, (2) Implementation of shared care, (3) Implementation of patient navigation, (4) Embracing emerging technologies, (5) Future focus on education, and (6) Future focus on nurse-led research.

## Introduction

There were 19.3 million new cancer cases, and 9.6 million cancer deaths occurred in 2023.^1^ Within the Australian context, over one million people are currently living with or beyond a diagnosis of cancer in Australia.^2^ Consequently, the impact of cancer causes a significant burden for health care systems across the entire cancer care continuum.^3^ Supportive care is a term used to describe a person-centred approach to the provision of the necessary multidisciplinary services for those affected by cancer to meet their psychological, physical, informational, spiritual, social needs during diagnosis, treatment, or follow-up phases, survivorship, palliation, and bereavement.^4^^,^^5^

Within the speciality of genitourinary (GU) cancers which includes penile, urothelial cancer (including bladder, ureter and urethral), prostate, testicular, adrenal and kidney cancers evidence has underscored that not all patients are reviewed by a multidisciplinary team (MDT), with a distinct lack of patient engagement in the process.^6^ Furthermore, a series of systematic reviews have identified that patients report a number of supportive care needs in penile,^7^ bladder,^8^ prostate,^9^ testicular,^10^ and kidney cancers.^11^ Oncology nurses provide a central contribution within the MDT and are well positioned to address the well-documented gaps by optimising supportive care.^12^, ^13^, ^14^ However, cancer nursing face an imminent care crisis internationally due to a shortage of nurses stemming from chronic recruitment and retention issues^15^ with evidence pointing out that up to 60% of cancer nurses will leave the profession within the next 10 years^16^ which will directly impact patient safety and missed nursing care.^17^

Within Australia there is an increasing need to delivering optimal care pathways and improve patient and health system outcomes particularly for regional/rural communities and disadvantaged populations.^18^ This is achieved through efficient, effective, and sustainable models of care that bridges private and public services with ongoing leadership in cancer nursing education.^18^

Most GU oncology nurses receive limited exposure to cancer care education in their undergraduate curriculum studies. They must complete many hours of non-specialist mandatory hospital training annually, with little time to keep abreast of the latest evidence-based developments in their speciality cancer field.^18^ Given the well-documented unmet supportive care needs among patients living with GU cancers^4^^,^^7^, ^8^, ^9^, ^10^, ^11^^,^^19^ capturing GU specialist nurses views on current gaps in service provision (private and public health systems), GU cancer nurses specific educational needs, and future priorities for research in the Australian setting is an important first step. Notably, in the Australian context there is a scarcity of universal oncology nursing specialisation programmes, including for GU cancers, and this observation is also reflected on a global scale.^20^ Therefore, this study sought to understand gaps in cancer service provision for patients affected by GU cancers, educational priorities for GU oncology nursing, and future priorities for research from the perspectives of GU nurses across Australia. This study addressed the following research question:

• What are the unmet needs for patient care, priorities for education and research among nurses providing care to people affected by GU cancers in Australia?

## Methods

### Design

A concurrent mixed methods study^21^ was conducted. This approach was taken whereby quantitative and qualitative data were collected simultaneously and analysed together. Firstly, a quantitative national survey of cancer nurses who were members of Cancer Nurses Society of Australia (CNSA) and the Australia and New Zealand Urological Nurses Society (ANZUNS) to explore self-perceived views on unmet care gaps, and priorities for education and research among nurses providing care to people impacted by GU cancers in Australia. Exploring participants' views in depth through semi-structured interviews helped to explain descriptive quantitative results by providing depth to the specific indications for gaps in care, and future priorities for education and research with the clinical rationale behind them. The final analysis and interpretation considered the interaction between the quantitative and qualitative findings.^21^ The rationale for comparing the quantitative and qualitative components were for a more comprehensive understanding of the research topic.

### Quantitative data collection instrument

An online survey was developed by the members of the GU Cancer Nurses Specialist Network Committee of the CNSA. The instrument consisted of an online questionnaire which included demographic and professional questions related to the self-perceived views on unmet care needs, and priorities for education and research which were free-text (Supplementary file 1). The quantitative descriptive survey was hosted using an online electronic data capture system (i.e., SurveyMonkey®), and the electronic link was sent to the CNSA who then sent an email invitation to the 1300 members and ANZUNS 1000 members. A reminder email was sent after two weeks by CNSA and ANZUNS. Participation was completely voluntary, and consent was assumed upon completion of the questionnaire. Data was collected anonymously without directly identifying information.

### Demographic and professional questions

The demographic and professional questions included location of workplace (metropolitan, regional, rural), participants' state and territory, private or public practice, cancer speciality, current clinical area of practice, gender, age, registered nursing experience (in months and years), oncology nursing experience (in months and years), highest qualification, and professional memberships.

The free text option for the participants centred around the questions of: (1) Can you nominate five or as many as you can top unmet supportive care needs for patients affected by GU cancers? (2) Can you please nominate five or as many as you can top educational needs in caring for people affected by GU cancers? (3) Can you please nominate five or as many as you can top nursing research priorities in caring for people affected by GU cancers? At the end of the survey, all the participants were asked a further question to elicit if they would be willing to take part in a semi-structured interview. If they were agreeable, they were asked to provide their names and contact telephone numbers so that members of the research team could contact them to arrange a suitable time to conduct the interview.

### Qualitative data collection

Semi-structured interviews were conducted with six cancer nurses involved in providing care for people affected by GU cancers. The numbers were lower than expected, however, all nurses who were agreeable to be interviewed were. The interviews were conducted by the researchers via Microsoft Teams during May to July 2022. A one-on-one semi-structured interview approach ensured that topics relevant to the research question were addressed, and lasted approximately 45–60 min which was flexible enough to enable participants to volunteer information on topics relevant to them, see Table 1 for examples of interview topic guide questions. All interviews were recorded and transcribed. These data were then compiled into a documentary record and rendered anonymous during data analysis. The researchers who conducted the interviews were all female, cancer nurses, with expertise in GU cancers, one of which included a professor of cancer nursing with expertise in mixed methods studies. None of the researchers had any previous relationships with the participants who were interviewed.

**Table 1:** Interview probe questions.

• **I am really interested in your experiences in caring for people affected by genitourinary cancers. I'm here today to hear about your experiences so:** • Can you tell me what you think are the unmet supportive care needs of people affected by GU cancers? ○ Can you tell me about your current habit/practice? ○ Can you tell me about other practice? ○ Can you tell me what are the barriers/facilitators to addressing unmet needs? ○ Specific prompts of areas of need (physical, psychological, family, intimacy, social, practical daily living, spiritual/existential, informational, cognitive and communication needs) among people affected by GU cancers? ○ Can you tell me how nurses provide care to GU patients across private and public sectors in health care? ○ Can you tell me how do you address the person-centred care needs of people affected by GU cancers to plan care? Intervene? And evaluate? patient care. ○ What would help in developing shared care plans with patients in your place of work? • Can you tell me what you think are the key educational needs in caring for people affected by GU cancers? ○ Probe (prostate, bladder, kidney, testicular, penile) • Can you tell me what you think are the key research priorities in caring for people affected by GU cancers? ○ Probe (prostate, bladder, kidney, testicular, penile)

GU, genitourinary.

### Sample

The Australian Institute for Health and Welfare (AIHW)^22^ identified that there are approximately 283,570 nurses working in Australia, inclusive of enrolled nurses, midwives, and registered nurses. Of these, an estimated 24,333 work within the medical division of their hospital. At the time of conducting this research there was no available information to determine the number of nurses who worked within the cancer speciality. The AIHW Labour Force data did not record the number of nurses who worked in cancer care, nor was the register of nurses in Australia available for this research purpose. It has only been in 2021 that the Australian Health Practitioner Regulation Agency (AHPRA), included the cancer nursing speciality as a subgroup of the nursing profession. Within the context of GU cancer nurses, the Australian Government currently funds 100 Prostate Cancer Foundation of Australia Nurses across the whole of Australia, and there are not designated funded specialist nurses for all other GU tumours. Therefore, the CNSA and ANZUNS was used as the point of collection of data nationally. The CNSA and ANZUNS are Australian wide professional bodies for all cancer nurses.

### Inclusion criteria

All nurses who self-identify to provide care to people affected by GU cancers in Australia. The inclusion criteria included registered nurses involved in direct care of people affected by GU cancers.

### Data management and analysis

Ethical approval was obtained from the University of Canberra (IRB No. 9182) and the approval from the Research Committee of the Cancer Nurses Society of Australia prior to participant recruitment and data collection. The survey administrator used properties of the survey system to download de-identified survey results into Microsoft Excel® spreadsheets that were given to the principal investigator (CP). The data was downloaded from the electronic platform, checked for accuracy, and stored on a password protected computer. A power analysis for the sample size was not calculated because there are no comparable studies published at the time of the study (to the best of our knowledge) and given the qualitative nature of the open-ended question. Descriptive statistics were computed to describe the study sample.^23^ Participants responses to the free-text questions relating to the educational priorities were analysed using content analysis.^24^ The participants' free-text responses were generally given using a single word descriptor relating to the educational topic that was of interest to them. These responses, identifying the priorities were read and coded multiple times by the research team to identify common keywords, synonyms, and similar terms. To analyse the qualitative interview data, thematic-analysis^25^ was conducted after checking saturation of information, the researchers read and re-read the transcribed data to identify themes until consensus reached. The researchers familiarized themselves with the data by reading the transcribed verbatim several times. Starting with line-by-line coding, statements related to unmet needs, and educational and research priorities which were coded and categorized. Specifically, the following steps were taken: familiarisation of the data; identifying a thematic framework; indexing themes; charting; mapping and interpretation.

Data integration during the analysis of the qualitative and quantitative data was important to provide a comprehensive understanding of the area. Specifically, this process involved merging the data into one dataset in excel file in tabular format and qualitising the quantitative data into narrative descriptions. The qualitative and quantitative data were connected during the data integration under the main areas of patient care, education, and research. Finally, this process involved data triangulation by cross-verifying the data from the survey and the qualitative themes to elucidate similarities and differences.

Trustworthiness and rigour were considered.^26^ Credibility was ensured by peer debriefing in the research team during the analysis process. Dependability was ensured by having a debriefing session among the interviewers (CP, DS, MR) to maintain consistency in data collection. All the interview transcripts were quality checked for accuracy. Confirmability was considered by keeping an audit trail throughout the process, including documenting post-interview reflective notes, and during the analysis to make an accurate record of all the decisions and processes undertaken during the study. Quantitative and qualitative data were analyzed separately and integrated together. Then, the results of both methods were compared to determine the degree to which they converged and diverged.

## Results

### Sample

A total of 50 nurses responded to the survey (Table 2). Females made up 78% of participants and most participants were over 40 years old. The sample included representation from all Australian states and territories, except for Northern Territory. The participants reported to have a wide variety of nursing role titles which included: Clinical Nurse Specialist (*n* = 8), Radiation Therapy Nurse Unit Manager (*n* = 1), Cancer Care Coordinator (*n* = 3), Associate Nurse Unit Manager (*n* = 1), Clinical Nurse Specialist in GU Cancers (Radiation Oncology) (*n* = 2), Prostate Cancer Specialist Nurse (*n* = 9), Manager of Nursing Program (*n* = 1), Clinical Nurse Consultant (*n* = 5), Case Manager (*n* = 1), Cancer Advisor (*n* = 1), Registered Nurse (*n* = 2), Clinical Urology Nurse (*n* = 2), Urology Nurse Practitioner (*n* = 3), Clinical Nurse Educator (*n* = 1), Endorsed Enrolled Nurse (*n* = 2), and GU Nurse Navigator (*n* = 1), and seven participants did not report their role title. Across the 50 nurse participants 38/50 (76%) provided care to people affected by prostate cancer, 29/50 (58%) bladder, 23/50 (46%) kidney, 19/43 (44%) testicular and 14/50 (28%) penile cancer in their current roles. A total of six participants consented to take part in a semi-structured interview. Five nurses were educated to master's level, one to bachelor's degree, five out of the six participants were females, and they all worked across private, public, metropolitan, regional and rural health care settings. All nurses who consented to take part in the interview process were interviewed.

**Table 2:** Distribution of the participant characteristics.

Characteristics	*n* = 50	%
**Gender**		
Female	39	78
Male	4	8
Missing	7	14
**Age category (years)**		
18–29	1	2
30–39	8	16
40–49	9	18
50–59	16	32
60 and over	10	20
Missing	6	12
**State or territory**		
Queensland	7	14
New South Wales	7	14
Australian Capital Territory	1	2
Victoria	18	36
South Australia	3	6
Western Australia	7	14
Northern Territory	0	0
Tasmania	1	2
Missing	6	12
**Location of practice**		
Metropolitan	35	70
Rural	9	18
Remote	0	0
Missing	6	12
**Type of service organisation**		
Public	32	64
Private	10	20
Both	2	4
Other	0	0
Missing	6	12
**Cancer specialty**		
Medical oncology	8	16
Radiation oncology	7	14
Surgical oncology	6	12
Combined	22	44
Missing	7	14
**Current area of practice**		
Inpatient	6	12
Outpatient	23	46
Ambulatory care	4	8
Education	1	2
Research	1	2
Administration	1	2
Other	8	16
Missing	6	12
**Years of GU cancer nursing experience**		
< 2 years	9	18
2–5 years	8	16
6–10 years	11	22
11–20 years	12	24
> 21 years	4	8
Missing	6	12
**Highest qualification**		
Hospital certificate	1	2
Diploma	1	2
Bachelor	8	16
Masters	12	24
Doctorate	0	0
Other	3	6
Missing	25	50

GU, genitourinary.

### Unmet supportive care needs

Overall, the nurses' responders perceived that the highest unmet supportive care needs were experienced in bladder [9 (23%)], penile [6 (26%)], prostate [5 (21%)], kidney [2 (8%)], and testicular [1 (4%)] cancer groups. Noteworthy, 27 participants did not answer this question so some caution should be taken in the interpretation of this data. Specifically, nurses involved in the care of people affected by GU cancer perceived that highest levels of unmet needs were psychological, intimacy followed by informational support, see Table 3. Specifically, the highest domains of perceived unmet needs related to psychological/emotional needs – 17/23 (74%), intimacy needs – 15/23 (65%) and informational needs – 13/23 (57%).

**Table 3:** Distribution of nurses perceived unmet supportive care needs.

Thinking about the GU cancer you have identified as having the most unmet supportive care needs (SCN).	No need (%, *n*)		Some need (%, *n*)		
	Not applicable	Satisfied	Low need	Moderate need	High need
1. Physical needs (*n* = 22) (experience of physical symptoms such as fatigue, pain, management of bladder voiding, etc.)	0%, 0	14%, 3	9%, 2	45%, 10	32%, 7
2. Psychological/emotional needs (*n* = 23) (experience of psychological/emotional symptoms such as anxiety, depression, worry, despair, fear, etc.)	0%, 0	4%, 1	0%, 0	22%, 5	74%, 17
3. Family related needs (*n* = 23) (experience of fears/concerns for the family, dysfunctional relationships, etc.)	0%, 0	4%, 1	0%, 0	65%, 15	30%, 7
4. Intimacy needs (*n* = 21) (sexual function, experience of fears/concerns for the family, dysfunctional relationships, etc.)	0%, 0	0%, 0	10%, 2	62%, 13	29%, 6
5. Social needs (*n* = 23) (experience of reduced social support, social isolation, loneliness, etc)	0%, 0	9%, 2	9%, 2	57%, 13	28%, 6
6. Practical needs (*n* = 23) (situations of transportation, out-of-hours access to health care, financial/economic support, etc)	0%, 0	13%, 3	9%, 2	65%, 15	13%, 3
7. Daily living needs (*n* = 23) (experience of restriction in daily living tasks such as exercise, housekeeping, etc)	0%, 0	9%, 2	26%, 6	57%, 13	9%, 2
8. Spiritual/existential needs (*n* = 23) (existential concerns such as fear of death, death and dying, fears regarding after life, etc)	0%, 0	4%, 1	4%, 1	78%, 18	13%, 3
9. Informational needs (*n* = 23) (experience of a lack of information, uncertainty of follow-up care, lack of information in relation to treatment and diagnosis, etc)	0%, 0	13%, 3	4%, 1	26%, 6	57%, 13
10. Patient-clinician communication needs (*n* = 23)(quality of communication between patients and health care professionals, satisfaction with care, shared decision-making, etc)	0%, 0	13%, 3	9%, 2	52%, 12	26%, 6
11. Cognitive needs (*n* = 22) (experience of cognitive impairments, memory loss, etc.)	5%, 1	5%, 1	50%, 11	32%, 7	9%, 2

GU, genitourinary.

In keeping with the nurse reports of patients perceived unmet needs in the quantitative survey, nurses articulated very similar priority gaps in supportive care for people diagnosed with GU cancers. Many of the nurses expressed concerns about a lack of a designated cancer specialist nursing program for patients as a central point of contact which should be tumour specific. Due to the fragmented nature of GU cancer services nurses observed a ‘*gap*’ in ‘*who*’ takes ‘*ownership*’ of the coordination of care and support for the patient and their significant other. Fragmented care co-ordination was also further negatively impacted due to distinct oncology and urology clinical nurses with different service managers, which negatively impacted clear communication in the MDT involved in providing GU cancer services. All the nurses spoke of the need to develop networks both locally and nationally to provide improved care co-ordination.

*“this really requires an ‘active effort’, that's the key term, of what it is you're actively engaging patients around intimacy, things in recovery of continence, exercise physiology, smoking cessation to provide care-coordination”*.

Nurses clearly articulated many issues that people living with GU cancer face within the Australian health system which included: (1) inadequate informational support across the cancer care continuum, (2) disparities between public and private cancer services with limited access to allied health support teams (psychologist, nutrition, social worker, physiotherapist), (3) profound shortage of psychological support in both public and private settings, (4) observed patient difficulty accessing the surgeon in private practice which is unlike public services, as there is no, or very limited access to registrars in private practice out-of-hours, (5) no mainstream support services for financial toxicity, and (6) a clear lack of Enhanced Recovery After Surgery Programs in the Australian context.

Several of the nurses also spoke about interprofessional differences in patient-clinician consultations which may contribute to difficulties for patients. Nurses spoke about concerns with consultants at the time of ‘breaking bad news’ of a new cancer diagnosis, and the profound gaps in survivorship care when cancer specialist nurses were not involved in the MDT.

*“… but surgeons don't seem to realise it's a real need in some cases, and they'll (surgeon) think they've explained things to the patients, and the patients know what's going on, but they really, really don't.”*

*“Surgeons in general maybe think well your life's been saved … and consequences of surgery are a small price for saving your life, but I'm not sure that they really get the whole psychological impact and importance of optimising survivorship care”*.

*“… it's disjointed, fragmented, you know, often patients can get lost in the system.”*

There was a notable perception among the nurses that supportive care was more accessible and enhanced patient outcomes in the public health care settings compared to the private health care setting.

*“I would argue that the care in public is … better and easier obtained by patients, because there's not an ownership or a don't go near my patient in the private, in the public nurses can go to an outpatient clinic and go through the lists, or outpatient list, or look at a theatre list and identify patients having biopsies, for instance, or see a patient that's diagnosed and booked for radical orchidectomy or something like that. So the access to patients I think, is better or easier in the public”*.

While all the nurses valued the central importance of validated supportive care needs screening tools to develop shared care plans to address unmet needs of people diagnosed with GU cancers, there was a lack of implementation at large in practice.

*“if you try and talk about a care plan, and if you wanna add another piece of paper that someone is being asked to fill in, a nurse has to fill out, you're gonna get … come up against resistance … ”*

*“Unfortunately, we're really bad at that. Like we have lost a lot of these, like standard questionnaires that you would think we should have. The only true tool that we use at the moment would be a distress thermometer. And like, unfortunately, in our centre that is filled up at point of registration. So, they before they actually meet before they actually have their first medical appointment. So, it's usually less useful for us who don't, this will sound like an excuse, but just purely capacity and workload, we haven't been able to really figure out a different way to address that problem.”*

### Educational needs

Nurses articulated similar priority areas to advance knowledge and professional development of the current and future GU cancer nursing workforce, see Table 4. Nurses expressed that the current GU cancer nursing workforce spanned from very junior nurses, to experienced GU nursing practitioners and nursing leaders, all of whom had differing knowledge and experience. Nurses spoke about the need for future educational resources and learning opportunities to be GU cancer specific, rather than generic cancer educational offerings. Educational offerings should be targeted to addressing the survivorship issues of people affected by GU cancers.

*“Probably one of the biggest barriers is not understanding what the needs are for these patients. If you don't understand what they are, then you can't ever address them properly.”*

**Table 4:** Nurse perceptions of priority educational topics, patient care, and research.

**Nurse perceptions of priority unmet care needs among patients affected by GU cancers**				
Priority 1	Priority 2	Priority 3	Priority 4	Priority 5

➢ Poor psychological care and access to specialist services ➢ Lack of tailored informational support ➢ Lack of support for relationships and intimacy support ➢ Significant financial toxicity in the Australian health care system ➢ Lack of shared care plans and supported self-management	➢ Better access to sexual/fertility counselling ➢ Sustainable social support in the community ➢ Improved care co-ordination (private and public services) ➢ Improved physical symptom support ➢ Assistance with social needs	➢ Support for changes in body image ➢ Improved continence management ➢ Improved support for lifestyle changes ➢ Improved communication between hospital cancer services and community providers ➢ Lack of emotional support for family members	➢ Poor access to support groups (e.g., bladder cancer) ➢ Poor communication between patients and their consultants ➢ Lack of community awareness of the less common types of GU cancers ➢ Lack of support for daily living ➢ Lack of availability for treatment-based rehabilitation and/or exercise programmes	➢ Lack of support for the development of patient self-efficacy ➢ Improved assistance with self-catheterisation for patients ➢ Lack of survivorship care planning support ➢ Lack of financial information
**Nurse perceptions of priority educational development topics needs among GU cancers**				
Priority 1	Priority 2	Priority 3	Priority 4	Priority 5
➢ Understanding the pathophysiology of GU cancers ➢ Understanding disease risk stratifications for GU cancers ➢ Education inclusive of all GU cancers not just prostate cancer ➢ Understanding patient pathways ➢ Understanding of rare cancers	➢ Understanding the community resources available ➢ How to communicate with patients with autism/intellectual disability ➢ How to access to equipment/incontinence pads ➢ Providing better emotional support for all GU cancers ➢ Regular updates on the latest therapies	➢ Understanding how to support the informal caregiver ➢ How to empower patients to be partners in their own care ➢ How to sign-post patients for financial support ➢ Side-effect profiles for all GU cancers ➢ Development of research skills and appraising evidence	➢ How to address issues with masculinity ➢ Supporting patients with lifestyle changes ➢ Understanding new and emergent therapies i.e. immunotherapy ➢ Understanding treatment options for all GU cancers ➢ Understanding COVID-19-related issues in cancer care	➢ Understanding psychosocial needs of all GU cancers ➢ Understanding informal caregiver experiences ➢ Understanding early role of palliative care ➢ How to manage late side effects of GU cancer treatments ➢ Understanding exercise recommendations
**Nurse perceptions of priority research areas among GU cancers**				
Priority 1	Priority 2	Priority 3	Priority 4	Priority 5
➢ Survivorship care needs, ➢ Psychological care, ➢ Unmet supportive care needs, ➢ Exploring intimacy and relationship experiences, ➢ Late effects	➢ Impacts of new and emergent treatments on patient experience, ➢ Impact of nurse-led services on patient outcomes, ➢ Patient experiences of treatment decisions, ➢ Caregiver experiences, ➢ Evaluation of holistic care experiences	➢ Future development of nurse-led interventions ➢ Experiences of care in private and public cancer services ➢ Impact of cancer stigma ➢ Understanding experiences of self-management ➢ Family impacts	➢ Patient experiences of living with untreated cancer i.e. active surveillance ➢ Impact of community-based cancer screening programmes ➢ Penile cancer needs as rare cancer ➢ Needs of people affected by bladder cancer ➢ Patient experiences of genetic testing	➢ Research focus on broad topics related to: ➢ Nurse-led services in GU oral agents ➢ (no further topics identified

GU, genitourinary.

Nurses also wanted to understand the pathophysiology of different GU cancers, understanding current and new emerging treatments, and addressing the psycho-social concerns across the entire cancer care continuum.

*“We need to remember we're treating the patient, not the cancer, I think it's extremely important that as nurses we have a good understanding of the pathophysiology of the disease and the treatments. They need to (know this) because those questions as much as you're treating the patient, if that patient asks a question, you need to be able to provide detailed and accurate information.”*

Nurses spoke about barriers to educational offerings which included reduced learning opportunities for specific GU cancers, and significant financial costs for training and professional memberships. Nurses reported that they would value educational study days which were inclusive of (1) consumers, (2) short self-directed learning packages specific for GU cancers (topics related to survivorship, palliative care, voluntary assisted dying), (3) understanding COVID-19 related issues, evidence-informed interventions of partners of those affected by cancer, further (4) leadership development for nurses to take a proactive approach in the MDT board meetings, and (5) in the adoption and implementation of the optimal care pathways in cancer.

*“A real lack of support is experienced, and you know I can only learn so much and educate myself so much about.”*

*“… it's easy to say that you have nursing teams in a multidisciplinary meeting, but, how much are they actually given a voice to, is definitely worth looking into.”*

### Research priorities

The nurses taking part in the interviews spoke less about the priorities for research among the GU nursing community. Research priorities were aligned to the priorities identified in Table 4 which included: (1) understanding the short- and long-term impact of cancer on partners and informal caregivers, (2) understanding the specific needs of people with rare cancers, and with particular attention to upper tract urothelial cancer and patients receiving intravesical therapies, (3) exploring future nursing interventions in relation to patient support for genetic testing and counselling, (4) developing interventions and support available to address financial toxicity, and (5) GU cancer nursing interventions for nurse-led geriatric oncology.

*“… understanding the lived experience so that we can then understand how we can better support them. Hearing the voice of the patient not presuming that we know what's affecting them, hearing from them, what's affecting them. And acknowledging that not everyone has the same experience, but looking for themes of what are these patients crying out for that they didn't understand? Or that, you know, if someone had just told me that this could happen, I wouldn't have fallen to pieces when it did happen.”*

*“… how do you develop interventions? Or how do you support patients if you actually don't know what their needs are?*

## Discussion

The aim of this research was to investigate the unmet needs for patient care, and priorities for education and research among nurses providing care to people affected by GU cancers. This explanatory sequential mixed methods study identified that the largest priority for GU nurses was the care needs of their patients. An experienced cohort of nurse participants reported that they valued that they had knowledge and skills to appropriately assess the needs of their patients to find active solutions across a fragmented, and often inequitable, health care system. Cancer care has become increasingly complex, because many are living longer with cancer and requiring greater capacity from the health system. Many nurses spoke of how they “fell” into their roles, it raises important concerns about lack of accessible structures to train and support future nurses to sustain GU cancer care services into the future^18^

The broader literature confirms that if health systems are fragmented and inequitable for patients, this increases morbidity and mortality risks.^27^ Nurse-led models have been successful, for example, in rural and remote models of patient care, with cancer nurses leading telehealth services.^28^ Cancer is rapidly evolving and nursing is well place to respond to care gaps in existing services, and it is essential that they have a seat at the table.^5^ Nurses are recognised as an antidote and the solution to cancer care,^29^ and there is clear evidence demonstrating that nurses can change cancer outcomes.^5^

As identified in our study, experienced nurses work within a broad scope of practice.^30^ Cancer nurses provide supportive care to people diagnosed with cancer, they are innovative in addressing gaps in health systems, support the broader clinical team, and provide education and training of the next generation of nurses.^17^ The nursing profession are observing a mass attrition of nurses from the workforce, particularly those with extensive clinical experience who have left the profession post-COVID-19.^31^ In a recent survey, 60% of nurses planned to leave the cancer workforce.^32^ For those that are staying, the risk of job satisfaction and burnout is likely.^33^ Known strategies to support nurses, such as clinical supervision, where nurses come together to share and debrief, are also not consistently available in a variety of settings.^34^

The Australian Government has recently increased its financial commitment to fund the future cancer nursing workforce to address many unmet needs as clearly articulated by the nurses represented in this study, and in keeping with the broader literature.^4^^,^^7^, ^8^, ^9^, ^10^, ^11^ There is growing appreciation that many more patients affected by cancer require timely and equitable access to cancer nurses.^35^ However, it was identified in our study that there is a lack of educational resources for cancer nurses which highlights future risks that can come if succession planning for workforce development is suboptimal. Education offerings requires a reliable and sustainable infrastructure, including consideration for undergraduate generalist cancer care preparation, similar to efforts being demonstrated in palliative care.^36^ In the context of the study findings, it was not surprising that research was a low priority in this study given the weight and importance placed upon patient care. However, further research is required to effectively develop workforce infrastructures that ensures cost-effective resources, protected time (for education and clinical research), and supportive and sustainable structures.

### Recommendations for practice

Given the prominence of addressing unmet cancer care needs among people with GU cancers in this study, cancer nursing as a discipline alongside the multidisciplinary team, requires innovative solutions to overcome fragmented care which is often highly complex, and develop individualised and integrated care across the cancer care continuum. We encourage clinicians, researchers, policy makers, people affected by cancer, and their care networks, to continue driving innovation accordingly with the following calls to action.

1. Embedding an integrated approach to cancer nursing. Cancer nurse leaders should advocate for integrated approaches to care planning, assessment, delivery, and evaluation in the nursing process and in planning future service development and delivery.^18^^,^^32^
2. Implementation of shared care models. Education, training and resources must be made available to all members of the cancer multidisciplinary team, primary care providers, people affected by cancer and their care networks to help them understand the value of shared care and their individual role that they play and contribute,^37^ including culturally safe care,^38^ and care of older adults with cancer.^39^
3. Implementation of patient navigation across the health system. Health service planners and policymakers must engage in robust workforce and program planning to determine how navigation support can be effectively implemented across the cancer care continuum.^40^
4. Embracing emerging technologies. There has been an increased uptake on emergent technologies in cancer, and careful and proactive considerations are required in how digital technologies can be, applied to facilitate innovations in cancer care while also considering potential practical and ethical pitfalls in cancer care delivery.^28^^,^^41^
5. Future focus on education. The future workforce needs to be equipped with the knowledge, skill and understanding to build the emotional intelligence, empathy, compassion, and capacity to care for people with cancer while protecting themselves from compassion burnout,^42^ inclusive of both undergraduate and post-graduate education.^18^
6. Future focus on nurse-led research. The continual emergence of new nursing roles opens opportunities for qualified nurses with academic talent to combine research, teaching, and practice simultaneously. Career pathways require infrastructure with resources to help sustain such pathways in the future. Future research should explore the contribution of oncology clinical academic nurses to improving patient outcomes and care delivery in an authentic and meaningful way to further develop and sustain research activity within the health care setting.^43^

### Limitations

This study was conducted with GU nurse members of the Cancer Nursing Society of Australia, and the Australia and New Zealand Urological Nurses Society (ANZUNS) located in Australia. The participants were drawn from a professional organisation for cancer nurses with a voluntary membership, which may have introduced bias. It is possible that these participants may be more committed to their career as cancer nurses compared to non-members, and may therefore, not be representative of the target population. The sample was biased in favour of females, nurses working in public hospitals, many who have worked on oncology of many years, were experienced and highly educated. This is an important consideration since younger and more inexperience nurses may report different educational and research priorities. Therefore, further research should be undertaken in this cohort of Australian cancer nurses. The nurses' responses to open-ended question were short, providing limited information about the type of education and what their preferences for the modality of educational delivery. Despite these limitations, the research team followed a transparent approach to improve the rigour, validity and confirmability of the findings throughout both the qualitative and quantitative aspects of the design study and conduct.

## Conclusions

Specialist GU cancer nurses support a broad group of patients. Given the prominence of addressing unmet cancer care needs among people with GU cancers in this study, cancer nursing as a discipline alongside the multidisciplinary team, requires innovative solutions to overcome fragmented care which is often highly complex, and develop individualised and integrated care across the cancer care continuum. We encourage clinicians, researchers, policy makers, people affected by cancer, and their care networks, to continue to drive innovation by (1) Embedding an integrated approach to cancer nursing, (2) Implementation of shared care, (3) Implementation of patient navigation, (4) Embracing emerging technologies, (5) Future focus on education, and (6) Future focus on nurse-led research.

## Ethics statement

Ethical approval was obtained from the University of Canberra (IRB No. 9182) and the approval from the Research Committee of the Cancer Nurses Society of Australia prior to participant recruitment and data collection. The research reported in this article has adhered to the relevant ethical guidelines. All procedures performed in studies involving human participants were in accordance with the ethical standards of the institutional and/or national research committee and with the 1964 Helsinki Declaration and its later amendments or comparable ethical standards. Informed consent was obtained from all individual participants included in the study.

## CRediT authorship contribution statement

**Catherine Paterson**: Conceptualization, Methodology, Data Collection, Validation, Interpretation, Writing Original draft, Writing, Reviewing & Editing, overall supervision. **Michelle Rosano**: Methodology, Data Collection, Interpretation, Reviewing & Editing. **Diana Schulz**: Methodology, Data Collection, Interpretation, Reviewing & Editing. **Helen Anderson**: Methodology, Interpretation, Reviewing & Editing. **Donna Cowan**: Methodology, Interpretation, Reviewing & Editing. **Kerry Santoro**: Methodology, Interpretation, Reviewing & Editing. **Tina Forshaw**: Methodology, Interpretation, Reviewing & Editing. **Cynthia Hawk**: Interpretation, Writing, Reviewing & Editing. **Natasha Roberts**: Interpretation, Writing, Reviewing & Editing. All authors had full access to all the data in the study, and the corresponding author had final responsibility for the decision to submit for publication. The corresponding author attests that all listed authors meet authorship criteria and that no others meeting the criteria have been omitted.

## Declaration of competing interest

The authors declare that they have no known competing financial interests or personal relationships that could have appeared to influence the work reported in this paper.

## Declaration of generative AI and AI-assisted technologies in the writing process

No AI tools/services were used during the preparation of this work.
