# Supplementary material for: What are the perceived unmet needs for patient care, education, and research among genitourinary cancer nurses in Australia? A mixed method study
Source: Asia Pac J Oncol Nurs. 2024 Jul 25;11(9):100564. doi: 10.1016/j.apjon.2024.100564 (PMC11403422; doi:10.1016/j.apjon.2024.100564)
Supplement: Multimedia component 1 [file mmc1.doc]

**[Administered via survey monkey]**

**Study:**

**What are the perceived unmet needs for patient care, education, and research among genitourinary cancer nurses?**

**Participant Consent**

| **1. Do you consent to participate in this study?** *(Compulsory Response)*  🞎 I consent to participate  🞎 I do not consent to participate |
| --- |

Demographics

| **Instructions:** The following questions relate to your background information. Please read carefully and write your response or select the most appropriate option. |
| --- |

| - 1. **What is your gender?** | ^1^🞎 Male | ^2^🞎 Female | | ^3^🞎 Other | | ^4^🞎 Prefer not to say |
| --- | --- | --- | --- | --- | --- | --- |
| - 1. **What is your age?** | 20-24 25-29 30-34 35-39 40-44 45-49 50-54 55-59 60-64 65-69 70-74  ................................................................................................................................ | | | | | |
| - 1. **In which state or territory do you live?** | 🞎 ACT  🞎 NSW  🞎 NT  🞎 QLD  🞎 SA  🞎 TAS  🞎 VIC  🞎 WA | | | | | |
| - 1. **What is your primary location of work?** | 🞎 Metropolitan | | 🞎 Rural | | 🞎 Regional | |
| - 1. **Do you work for a private or public organisation?** | 🞎 Private | | 🞎 Public 🞎Both | | | |
| - 1. **Which of the following best describes your current area of cancer practice?** | 🞎 Inpatient  🞎 Outpatient  🞎 Community  🞎 Theatres  🞎 Education  🞎 Research  🞎 Administration  🞎 Other: _________ (drop **down** for text) | | | | | |
| - 1. **What cancer speciality is your primary area of practice?** | 🞎 Medical oncology  🞎 Radiation oncology  🞎 Surgical oncology  🞎 Combined  🞎 Other: ____________ (drop down for text) | | | | | |
| - 1. **What is the highest level of qualification that you have completed?** | ^1^🞎 Hospital Certificate | | ^2^🞎 TAFE qualification | | | |
|  | ^3^🞎 Bachelor Degree | | ^4^🞎 Honours Degree | | | |
|  | ^5^🞎 Postgraduate Certificate | | ^6^🞎 Postgraduate Diploma | | | |
|  | ^7^🞎Masters Degree | | ^8^🞎 Masters Degree by Research | | | |
|  | ^9^🞎 Doctorate  (PhD or Professional Doctorate) | | ^10^🞎 Other, please explain: | | | |
| - 1. **Occupational role/title:** | ................................................................................................................................ | | | | | |
| - 1. **How long have you worked in GU cancers?** | 🞎Less than 2 years  🞎2-5 years  🞎6-10 years  🞎 11-15 years  🞎 16-20 years  🞎21-25 years  🞎 26-30 years  🞎 More than 31 years  .................................................................................................................... | | | | | |
| - 1. **Which of the following GU cancers are you involved with in your current nursing role? Tick as many as apply** | 🞎 Prostate  🞎 Bladder  🞎 Kidney  🞎 Penile  🞎 Testicular | | | | | |

| - 1. **Please tick which associations you are a member of (you may tick more then 1)** | 🞎 CNSA  🞎 ANZUNS  🞎 Other  Please specify |
| --- | --- |

**The following questions relate to your perceptions of unmet supportive needs among patients affected by GU Cancers:**

**Which GU Cancer do you think has the most unmet supportive care needs?**

🞎 Prostate

🞎 Bladder

🞎 Kidney

🞎 Penile

🞎 Testicular

| To help us understand how to better services for people diagnosed with GU cancer, we are interested in whether or not the needs of people affected by GU cancers are being met. For every item on the following page, indicate whether you perceive patients have had unmet supportive care needs within the last month in relation to the particular GU cancer you have identified as having the most unmet supportive care needs. |
| --- |

|  | **No need** | | **Some need** | | |
| --- | --- | --- | --- | --- | --- |
| **Thinking about the GU cancer you have identified as having the most unmet supportive care needs (SCN). Please complete the**  **following** | **Not applicable** | **Satisfied** | **Low need** | **Moderate need** | **High need** |
| 1. Physical needs   (Experience of physical symptoms such as fatigue, pain, management of bladder voiding, etc.) | 1 | 2 | 3 | 4 | 5 |
| 1. Psychological/emotional needs   (Experience of psychological/emotional symptoms such as anxiety, depression, worry, despair, fear, etc.) | 1 | 2 | 3 | 4 | 5 |
| 1. Family related needs   (Experience of fears/concerns for the family, dysfunctional relationships, etc.) | 1 | 2 | 3 | 4 | 5 |
| 1. Intimacy needs   (Sexual function, Experience of fears/concerns for the family, dysfunctional relationships, etc.) | 1 | 2 | 3 | 4 | 5 |
| 1. Social needs   (Experience of reduced social support, social isolation, loneliness, etc) | 1 | 2 | 3 | 4 | 5 |
| 1. Practical needs   (Situations of transportation, out-of-hours access to healthcare, financial/economic support, etc) | 1 | 2 | 3 | 4 | 5 |
| 1. Daily living needs   (Experience of restriction in daily living tasks such as exercise, housekeeping, etc) | 1 | 2 | 3 | 4 | 5 |
| 8. Spiritual/existential needs  (Existential concerns such as fear of death, death  and dying, fears regarding after life, etc) | 1 | 2 | 3 | 4 | 5 |
| 1. Informational needs   (Experience of a lack of information, uncertainty of follow-up care, lack of information in relation to treatment and diagnosis, etc) | 1 | 2 | 3 | 4 | 5 |
| 1. Patient-clinician communication needs   (Quality of communication between patients and healthcare professionals, satisfaction with care, shared decision-making, etc) | 1 | 2 | 3 | 4 | 5 |
| 1. Cognitive needs   (Experience of cognitive impairments, memory loss, etc.) | 1 | 2 | 3 | 4 | 5 |
| 1. Can you please nominate 5 or as many as you can top unmet supportive care needs for patients affected by GU cancers? | 1.  2.  3.  4.  5. | | | | |

| To help us understand what your educational needs are in caring for people diagnosed with GU cancer, can you please nominate education subjects that are important in your professional development. |
| --- |

| 1. Can you please nominate 5 or as many as you can top educational needs in caring for people affected by GU cancers? | 1.  2.  3.  4.  5. |
| --- | --- |

| To help us understand what are the research priorities in caring for people diagnosed with GU cancer, can you please nominate 5 top priorities for future research. |
| --- |

| 1. Can you please nominate 5 or as many as you can top nursing research priorities in caring for people affected by GU cancers? | 1.  2.  3.  4.  5. |
| --- | --- |

| 1. Do you have any other comments you would like to share? |  |
| --- | --- |

Thank you for taking the time to complete the questionnaire. The next section relates to participation in the qualitative interviews. If you would like to participate in an interview, you will be redirected to a separate survey, so that you can securely provide your email address. This separate survey will ensure that the results of your questionnaire remain anonymous. If you are selected, the interview will take approximately 45 minutes and will be conducted either online or via telephone. You will be asked a series of open-ended questions that explore your educational needs, perceived patient unmet supportive care needs and future priorities for GU cancer nursing research. Participation is completely voluntary. Thank you for your consideration.

**Interview Participation**

| **Would you like to participate in an interview?**  🞎 Yes (i*f a participant selects ‘yes’ they will be re-directed to a separate survey to collect contact information)*  🞎 No (i*f a participant selects ‘no’ they will receive a thank you message for their completion)* |
| --- |

***Separate Survey***

Contact Information

| **Thank you for choosing to participate in an interview. The questions you have already answered will not be linked to your contact information; your responses will remain completely anonymous.**  **In order to arrange an interview, please provide your work (or best contact) email address below:** | | | |
| --- | --- | --- | --- |
| 1. **Primary GU cancers you are involved with:** | ^1^🞎 Prostate | ^2^🞎 Bladder/urothelial | ^3^🞎 Testicular |
|  | ^4^🞎 Penile | ^5^🞎 Kidney |  |

**Thank you for taking the time to complete this questionnaire.**
